# Screening for autistic spectrum disorder in early childhood

**DOI:** 10.1186/s12887-021-02700-5

**Published:** 2021-09-08

**Authors:** Sophie Jullien

**Affiliations:** Barcelona Institute for Global Health, University of Barcelona, Barcelona, Spain

**Keywords:** Screening, Autism spectrum disorder, Preschool child

## Abstract

We looked at existing recommendations and supporting evidence on the effectiveness of screening young children for autistic spectrum disorder (ASD) for improving short- and long-term outcomes.

We conducted a literature search up to the 8th of November 2019 by using key terms and manual search in selected sources. We summarized the recommendations and the strength of the recommendation when and as reported by the authors. We summarized the main findings of systematic reviews with the certainty of the evidence as reported.

There are discrepancies among the recommendations given by different institutions on universal screening for ASD in children. Some recommend that all children should be screened with an ASD-specific instrument during well-child visits at ages 18 and 24 months in conjunction with ongoing developmental surveillance and broadband developmental screening; some conclude that the current evidence is insufficient to assess the balance of benefits and harms of screening for ASD in young children for whom no concerns of ASD have been raised by their parents or a clinician; and others recommend against universal screening, but for a screening among children with high risks.

There is adequate evidence that ASD screening tools applied to children between 12 and 36 months accurately identify those with ASD. There is some evidence showing benefit of early interventions applied to children with ASD, from children identified with developmental concern by their family, teacher or clinicians. We found no evidence on the effectiveness of interventions applied to children with ASD detected through screening.

## Background

### Introduction

The World Health Organization (WHO) European Region is developing a new pocket book for primary health care for children and adolescents in Europe. This article is part of a series of reviews, which aim to summarize the existing recommendations and the most recent evidence on preventive interventions applied to children under five years of age to inform the WHO editorial group to make recommendations for health promotion in primary health care. In this article, we looked at existing recommendations and supporting evidence on the effectiveness of screening young children for autistic spectrum disorder (ASD) for improving short- and long-term outcomes. We looked at the accuracy of the screening tests for detecting ASD, at the efficacy of existing interventions for children identified with ASD, and at the potential harms derived from ASD screening and the associated interventions.

### Why is screening for autistic spectrum disorder important?

Autism, or ASD is a developmental disorder and refers to ‘a range of conditions characterised by some degree of impaired social behaviour, communication and language, and a narrow range of interests and activities that are both unique to the individual and carried out repetitively’ [1].

ASD begins in childhood and becomes apparent during the first five years of life in most cases [1]. The aim of screening for ASD is to detect the disorder at an early stage for early interventions to reach better communication and social skills and overall quality of life for the affected people and their family.

### Context

There are uncertainties around the accuracy of the prevalence estimates of ASD. The global median prevalence of ASD was estimated at 6.2/10000 children in 2013, but estimates have increased up to 1 to 2% of the population [2–5]. Several factors have been identified as presumed causes for this growing prevalence, including improved awareness and services, the wider diagnostic criteria and the inclusion of milder cases, the improved and earlier detection of cases, the increased survival of children with serious disability, and environmental and socio-economic related factors [4, 5]. ASD is a lifelong neurodevelopmental disorder, associated with high burden. It was estimated that ASD represented 15.6% of disability-adjusted life years (DALYs) among all DALYs of the Spanish population aged 0 to 14 in 2013 [3].

In May 2014, 60 countries supported the resolution adopted during the 67th World Health Assembly on ‘Comprehensive and coordinated efforts for the management of autism spectrum disorders (ASD)’ [1]. While there is a global consensus supporting correct diagnosis and comprehensive management of ASD, whether universal screening for ASD should be implemented in all children is still debated. The screening tool to be used is also a matter of debate. An autism screening tool is defined as ‘a formalized brief questionnaire completed by a parent or provider before an in-depth diagnostic evaluation to identify a child at risk of autism’ [6]. There are two types of screening. The first one is the universal screening, or level 1 screening, which consists in screening ASD in the whole population, also referred as unselected population, or low-risk children. The second one is a selective screening, or level 2 screening, which consists of screening children with developmental concern, also referred as selected population or high-risk children. In this document, we will focus on universal screening.

## Key questions

1. How accurate are the screening tests for detecting ASD in early childhood?
2. What is the effect of interventions targeting young children identified with ASD in short- and long-term outcomes?
3. Does screening for ASD in young children improve short- and long-term outcomes?
4. What are the potential harms of ASD screening and interventions for children and their family?

## Search methods and selected manuscripts

We described the search methods, data collection and data synthesis in the second paper of this supplement (Jullien S, Huss G, Weigel R. Supporting recommendations for childhood preventive interventions for primary health care: elaboration of evidence synthesis and lessons learnt. BMC Pediatr. 2021. 10.1186/s12887-021-02638-8).

The search was conducted up to the 8th of November 2019, by manual search and by using the search terms “autism” and “autistic spectrum disorder”. From the WHO, we included two documents we considered relevant on this area. We found recommendations and their supporting evidence from the United States Preventive Services Task Force (USPSTF) (2016), PrevInfad (2017) and the American Academy of Pediatrics (AAP) (2015). We identified recommendations from the Centers of Disease Control and Prevention (CDC), and two guidelines from the National Institute for Health and Care Excellence (NICE) (although we did not find clear recommendation regarding universal screening from the latest).

The search in the Cochrane library by using the search terms “autistic disorder” or “autism spectrum disorder” in titles, abstracts or keywords returned 23 reviews and seven protocols. By screening the titles and abstracts, we included one review. While we identified additional reviews that address the effectiveness of different interventions for ASD (such as music therapy, communication interventions in minimally verbal children, interventions based on the Theory of Mind cognitive model, and parent-mediated early intervention), we did not include these reviews as we considered that they would not provide data on whether early application of the intervention would lead to better outcomes.

We included three additional manuscripts that we identified by contacting field experts or by hand search in the references of the already identified resources. Finally, we cite four manuscripts that we considered beyond the scope of this document but of potential interest for the readers of this article to complement the summary. The narrative review by Lord et al., recently published in the Lancet (2018), addresses ASD screening, early diagnosis and interventions (not summarised in the existing evidence section below due to the nature of the review).

All the included manuscripts for revision in this article are displayed in Table 1.

**Table 1:** Included manuscripts for revision

Sources	Final selected manuscripts
**WHO**	• WHO 2019 – (Fact sheet) [1] • WHO 2013 – Autism spectrum disorders and other developmental disorders. From raising awareness to building capacity. (Meeting report) [2]
**USPSTF**	• Siu 2016 – Recommendations [7] • McPheeters 2016 – Evidence support and systematic review [8]
**PrevInfad**	• 2017 recommendations and supporting evidence [3]
**CDC**	• Screening and diagnosis of autism spectrum disorder (website, recommendations) [9]
**AAP**	• Zwaigenbaum 2015 - Early Screening of Autism Spectrum Disorder: Recommendations for Practice and Research [10]
**NICE**	• NICE 2011 - Autism spectrum disorder in under 19 s: recognition, referral and diagnosis [11] • NICE 2013 - Autism spectrum disorder in under 19 s: support and management [12]
**UK NSC**	• UK NSC 2012 - ASD Policy Position Statement and summary [13]
**Cochrane Library**	• Reichow 2018 - Early intensive behavioral intervention for young children with autism spectrum disorders (Systematic review) [14]
**Other sources**	• Zwaigenbaum 2019 - Early detection for autism spectrum disorder in young children (Position statement from the Canadian Paediatric Society) [15] • Yuen 2018 – ‘Assessing the accuracy of the Modified Checklist for Autism in Toddlers: a systematic review and meta-analysis’ [16] • Soto 2014 – ‘A review of cultural adaptations of screening tools for autism spectrum disorders’ [6]
**Further reading**	• Lord 2018 – Autism spectrum disorder (Narrative review) [5] • Dow 2019 - Screening for autism spectrum disorder in a naturalistic home setting using the SORF at 18–24 months [17] • Bejarano-Martín 2019 - Early Detection, Diagnosis and Intervention Services for Young Children with Autism Spectrum Disorder in the European Union (ASDEU): Family and Professional Perspectives [18] • Salomone 2015 - Use of early intervention for young children with autism spectrum disorder across Europe [19]

**Abbreviations:** AAP: American Academy of Pediatrics; CDC: Centers for Disease Control and Prevention; NICE: National Institute for Health and Care Excellence; PrevInfad: PrevInfad workgroup from the Spanish Association of Primary Care Pediatrics; UK NSC: UK National Screening Committee; USPSTF: US Preventive Services Task Force; WHO: World Health Organization

## Existing recommendations

We summarized the existing recommendations and the strength of recommendations as per their authors in Table 2.

**Table 2:** Summary of existing recommendations

Source	Ref	Date	General recommendations for autism screening in children under five
**WHO**	[1]	2018	“Intervention during early childhood is important to promote the optimal development and well-being of people with an ASD. Monitoring of child development as part of routine maternal and child health care is recommended.”
**USPSTF**	[7]	2016	“The USPSTF concludes that the current evidence is insufficient to assess the balance of benefits and harms of screening for ASD in young children for whom no concerns of ASD have been raised by their parents or a clinician. (I statement)”
**PrevInfad**	[3]	2017	• “Not to do universal screening with tests like M-CHAT (M-CHAT, M-CHAT/F, M-CHAT/R, M-CHAT/R/F) is suggested.” (Low quality of the evidence; weak recommendation) • “Screening with tests like M-CHAT/R/F (M-CHAT, M-CHAT/F, M-CHAT/R, M-CHAT/R/F) in high risk individuals is recommended” (Moderate-high quality of the evidence; strong recommendation) High risk individuals: Familiar history of ASD in siblings, neurological disorders associated to ASD, prematurity, social communication disorders or repetitive behaviour or alert signs of ASD.
**CDC and AAP**	[9]	2015	“All children should be screened specifically for ASD during regular well-child doctor visits at: 18 month and 24 months. Additional screening might be needed if a child is at high risk for ASD (e.g., having a sister, brother or other family member with an ASD) or if behaviors sometimes associated with ASD are present.”
**UK NSC**	[13]	2012	“A national screening programme for autistic spectrum disorders in children under the age of five is not recommended”
**Canadian Paediatric Society**	[15]	2019	“All Canadian children should be monitored for early behavioural signs of ASD as part of general developmental surveillance.” “However, because randomized clinical trials have not yet demonstrated that routine developmental screening for children with no preidentified risks improves outcomes, the Canadian Task Force on Preventive Healthcare has concluded that the evidence is insufficient to recommend routine screening”

**Abbreviations:** AAP: American Academy of Pediatrics; CDC: Centers for Disease Control and Prevention; CHAT: Checklist for Autism in Toddlers; PrevInfad: PrevInfad workgroup from the Spanish Association of Primary Care Pediatrics; UK NSC: UK National Screening Committee; USPSTF: US Preventive Services Task Force; WHO: World Health Organization

## Existing evidence

The USPSTF commissioned a systematic review ‘to evaluate the evidence on the accuracy, benefits, and potential harms of brief, formal screening instruments for ASD administered during routine primary care visits and the benefits and potential harms of early behavioural treatment for children identified with ASD through screening’ [7, 8]. The literature search was conducted up to August 2014, and focused on including studies of ASD screening in children between 12 and 36 months who were unselected, that means asymptomatic children with no particular risk factor for ASD or without already identified concern about potential developmental delay [7].

The document from the PrevInfad group focused on children between 12 and 24 months [3].

### Accuracy of the screening tests for detecting ASD in children

Several screening tools are available for detecting ASD in children younger than 30 months.

The USPSTF review conducted by McPheeters et al. assessed the performance characteristics of tools for screening ASD in children between 12 and 36 months of age, and found adequate evidence that currently available screening tests can detect ASD among those children [7, 8].

The PrevInfad document summarised the literature available in this topic, including the findings from the USPSTF review [3].

A review on the evidence for ASD screening was performed by Zwaigenbaum et al. to support the recommendations developed by the AAP [10]. Based on a literature search updated in December 2013, the working group summarized published research on screening tools developed for use in children under the age of 24 months. They reached the overall statement that ‘evidence supports the usefulness of ASD-specific screening at 18 and 24 months’ and that ‘ASD screening before 24 months may be associated with higher false-positive rates than screening at ≥24 months but may still be informative.’

Recently, the Canadian Paediatric Society has published a document with their position statement [15]. The document provides a clear and short review of the literature and current state of evidence for screening ASD in children.

We also report findings on the performance of the screening tools in high risk children, and the effect of age as well as translation and cultural adaptation in the performance of these tools.

### Accuracy of the different screening tools

#### CHAT, M-CHAT, M-CHAT/F and M-CHAT-R/F

The Checklist for Autism in Toddlers (CHAT) tool was the first tool developed for universal screening of ASD in children [10]. It was found to present low sensitivity for universal screening [8, 10] and has been replaced by the Modified Checklist for Autism in Toddlers (M-CHAT) and its subsequent revisions [3, 7]:

- Modified Checklist for Autism in Toddlers (M-CHAT), 23 items.
- Modified Checklist for Autism in Toddlers-Revised (M-CHAT-R), reduced version of 20 items.
- Modified Checklist for Autism in Toddlers with Follow-Up (M-CHAT-F), 23-items version followed by an interview with the parents.
- Modified Checklist for Autism in Toddlers–Revised with Follow-Up (M-CHAT-R/F), reduced version of 20 items followed by an interview with the parents.

The M-CHAT and subsequent versions are the most used and most studied screening tools for ASD in children. They were designed to screen ASD in children between 16 and 30 months, and assess communication skills, joint attention, repetitive movement, and pretend play. It is based on a parent-rated scale that can lead to a follow-up interview. If the screening is positive, the child is referred for confirmatory diagnosis.

McPheeters et al. identified two good- and four fair-quality studies (in overall nine publications) that assessed the use of the M-CHAT and the M-CHAT/F in unselected children between 12 and 36 months of age [8]. Zwaingenbaum et al. identified and summarized eight studies that assessed these screening tools, coinciding with the seven publications already included in the review by McPheeters et al. [10]. The largest study and one of those judged as good-quality study (Chlebowski 2013) was conducted in the US, included over 18,000 children between 18 and 30 months of age, and found a positive predictive value (PPV) of 54% when using M-CHAT/F [20]. Estimates of sensitivity and specificity could not be determined from this study.

Both systematic reviews [8, 10] identified another large good-quality trial (Robins 2014) conducted in the US that assessed the performance of M-CHAT-R/F in 16,115 children between 16 and 31 months [21]. The M-CHAT-R/F showed a PPV of 48%, similar to the M-CHAT/F tool [3, 8, 10]. Similarly to the large Chlebowski study, sensitivity and specificity estimates could not be determined from this study. However, according to McPheeters et al., the M-CHAT-R/F tool was associated with a higher performance to detect children with ASD than the M-CHAT/F tool (67 per 10,000 vs. 45 per 10,000; *p* = 0.003) [8].

Both trials presented high attrition rates [20, 21]. ‘The validity of these two studies was weakened somewhat by the high dropout rate between screening steps, but was still reasonably high for mass screening’ [7].

Yuen et al. conducted a systematic review with the aim ‘to summarize the accuracy of the M-CHAT in children screened for ASD and to quantify the extent to which these measures of accuracy change in relation to the age at screening, sex distribution, study design, and background risk for ASD’ [16]. The literature search was conducted up to May 2018 and identified 13 studies for inclusion in the review. They were conducted in the US (*n* = 9), Canada (*n* = 2), Singapore (*n* = 1) and the UK (*n* = 1). Children were screened with M-CHAT at 21 to 41 months of age, and the diagnostic assessment of ASD was performed at 24 to 52 months. Three studies assessed the performance of M-CHAT in children with no developmental concerns (low-risk children), and 12 studies on selected children with developmental concerns (high-risk children).

The pooled sensitivity of M-CHAT to detect children with ASD was 83% (95% confidence interval [CI] 75 to 90). Sensitivity was higher when screening was performed at 30 months of age, compared to 24 months. The pooled specificity was 51% (95% CI 41 to 61), and specificity was comparable across different ages at screening.

The review authors found that there was a lack of evidence on the performance of M-CHAT in low-risk children (PPV of 6%; 95% CI < 1 to 14), and that M-CHAT accuracy for detecting ASD among high-risk children was low to moderate (PPV of 53%; 95% CI: 43 to 63) [16].

#### First year inventory

The First Year Inventory (FYI) is a tool designed to identify ASD in 12-month-old children. It is based on a parent-report questionnaire that consists of 63 items on social-communication and sensory-regulatory domains.

Both the USPSTF and the AAP reviews identified two studies that assessed this screening tool among children at 12 months of age in the US (*n* = 699) and in Israel (*n* = 583) [8, 10]. These two studies were considered of fair quality by McPheeters et al. Attrition rate was very high (82%) between children identified at high risk of ASD with the screening tool and those who underwent the diagnostic assessment. Performance characteristics were not provided due to the high attrition rate.

#### Early screening of autistic traits questionnaire

The Early Screening of Autistic Traits Questionnaire (ESAT) is a tool to identify ASD in children aged between 14 and 15 months in combination with specific developmental surveillance. It is based on social development and play behaviour, and consists in a two-stage screening: ‘children are prescreened with a four-item version of the ESAT at well-child visits; subsequently, for children screening positive on the four-item measure, a 14-item version of the questionnaire is completed by a home behavioural professional with parental input’ [8].

Both the USPSTF and the AAP reviews identified the same one fair-quality study that assessed the ESAT tool among 31,724 children aged between 14 and 15 months in the Netherlands [8, 10]. This study found that the ESAT presented low performance to identify children with ASD, with a PPV of 25%. ‘Targeted clinical surveillance and concern identified more children (*n*=39) with ASD than use of the ESAT (*n*=18)’ [8].

#### Social attention and communication study

The Social Attention and Communication Study (SACS) measure is ‘an observational tool designed to be completed by maternal and child health nurses conducting well-visits with infants and toddlers’ [8]. It is based on the evaluation of social and communication developmental milestones at 8-, 12-, 18-, and 24-month during the well-child visits, and ‘children failing specific combinations of critical items at these specified time points are identified as at risk for ASD from 12 months onward’ [8].

McPheeters et al. identified one fair-quality study that assessed the SACS among 20,770 children between 8 and 24 months in Australia. The SACS identified 1% of children at high risk of ASD. Among them, attrition rate was around 50%, and among those completing the diagnostic assessment, 81% were diagnosed with ASD and 18% with developmental delay or language disorder. There was no data on false negatives.

#### Young autism and other developmental disorders check-up tool

The Young Autism and Other Developmental Disorders Check-up Tool (YACHT) ‘consists of a developmental questionnaire (i.e., motor functioning, communication, social interaction), a caregiver interview regarding pointing and language comprehension, and a specific examination of children asking them to point to identified picture cards’ [8].

McPheeters et al. identified one fair-quality study that assessed the YACHT among 2814 children aged 18 months in Japan. The review authors concluded that ‘screening with elements of the YACHT as early as 18 months of age identified some cases of ASD within community samples of Japanese children’, but ‘little information is available about screen negatives’ [8].

#### Age of screening

In their review, McPheeters et al. looked at whether age at which ASD screening is performed modifies the performance characteristics of ASD screening tests [8]. Their findings are the following:

‘One study [22], including a subsample of high- and low-risk children also reported in other M-CHAT studies [20], attempted to examine screening characteristics of the M-CHAT at different ages but all within the very young age group of younger than 30 months. The researchers examined outcomes for low-risk children between 17 and 23 months of age (*n* = 4265; mean age, 18.57 months) and at 24 to 30 months of age (*n* = 1785; mean age, 24.74 months). PPV for children at older ages (61%) was better than the younger group (28%). Because this study had already excluded children who had previously been identified as being of concern for developmental delays, the performance characteristics are likely not reflective of what might be seen in the complete population. It also provides no data on screening children at preschool ages versus older ages. Data on false-negative results were unavailable.’

#### Cultural adaptations of screening tools

Most screening tools for ASD have been created in English in the US or in the UK. They are then translated and used in other settings [6]. However, ‘appropriate use of existing tools in other cultural and linguistic environments goes beyond translation to include a thorough process of identifying potential incongruities in language and concepts and then modifying the tool so that it is understood by the target population’ [6].

Soto et al. conducted a systematic review with the aim to ‘identify ASD screening tools that have been culturally adapted across cultures and countries; evaluate the extent to which the adaptation process adhered to recommended cultural adaptation guidelines, report on the psychometric properties of the adapted tools; and describe the implications of these findings for further research and practice [6]. The review authors included 21 studies that reported the results of the adaptation of nine different screening tools in eight different languages for children from 12 months to 18 years. For each included study, the review authors provide ‘ratings of the adaptation process of the screening tool used and the reported classification measures (sensitivity, specificity, PPV and NPV) of the screen for identifying children with ASD from those with typical development or other clinical conditions’. They found that ‘differences between the psychometric properties of the original and adapted versions were common, indicating the need to obtain normative data on populations to increase the utility of the translated tool.’

Aware of the potential variation of the performance of a translated screening tool for detecting ASD in children, the PrevInfad group reported the performance of the M-CHAT tool in Spanish. M-CHAT was translated, culturally adapted and validated in Spain with estimates of sensitivity (82%) and specificity (99%) similar to those from the original tool [3]. PPV was estimated at 38%.

#### False positives

False positives derived from the application of ASD screening tools refer to children identified at high risk of ASD that need to be referred for complete ASD diagnosis assessment, when those children truly do not have ASD.

Some working groups defend the universal screening of ASD in primary care despite the false positive rate, since they estimate that most false positive cases are affected by other developmental disorders that are equally subsidiary of referral to early intervention [3].

But other groups such as the PrevInfad group state that ‘massive screening of ASD in low risk population would produce an estimated positive predictive value around 38% in our setting, with an excess of referrals to specialised services and labelling effect on the patients’ and they consider it is more appropriate to screen ‘the population at risk or when concerns from parents or professionals are present’ [3].

### Effectiveness of interventions targeting young children with ASD in short- and long-term outcomes

At present, there is no cure for ASD. However, different interventions are available for children with ASD, such as behavioural, medical, educational, speech/language, and occupational therapy and complementary and alternative medicine approaches. These interventions are orientated to teach skills for daily life, reduce repetitive and obsessive behaviours, improve adaptive behaviours, and facilitate the acquisition of receptive and expressive language.

‘Treatments for young children in the target age group for routine screening for ASD are primarily behavioural interventions, particularly early intensive behavioural and developmental interventions’ [7]. These interventions may incorporate a commonly used modality called applied behaviour analysis (ABA), which consists of modifying inappropriate or undesirable behaviours, and promoting and reinforcing those that are most appropriate. This modality can be administered by trained clinicians or by the parents after a training period [3, 7].

**T**he WHO recognized that ‘evidence-based psychosocial interventions, such as behavioural treatment and parent skills training programmes, can reduce difficulties in communication and social behaviour, with a positive impact on wellbeing and quality of life for persons with ASD and their caregivers’ [1].

The review by McPheeters et al. provides a comprehensive summary of all interventions targeting young children with ASD on several outcomes [8]. We hereby report the main findings of the review. The authors identified 42 studies that addressed early intensive behavioural and development interventions for children with ASD, including 26 RCTs (nine good and 17 fair quality). Studies were generally very small, and ‘assessment of treatment evidence was complicated by the variation among studies in intervention design, method of delivery, comparators, and outcomes measured, as well as by the heterogeneity in the age, types of symptoms, and symptom severity of the children enrolled.’ The review authors considered that ‘data are inadequate to predict which children are most likely to benefit from early intervention, because benefits achieved differ by child characteristics and interventions offered’.

- Four randomized controlled trials (RCTs) (including the one included in the Cochrane review we describe below) reported cognitive and language outcomes for early intensive behavioural intervention (EIBI) delivered by trained clinicians [8]. Three of these RCTs (including the largest one, with 294 children) found that EIBI improved cognitive scores by 11 to 16 points (based on Mullen Scales of Early Learning or IQ) compared with a range of comparators, while the fourth RCT found no effect of EIBI. Authors found similar patterns for language outcomes.
- Twelve RCTs of play- or interaction-based interventions reported ‘significant improvements in some measures of interaction but not others’ [7, 8].
- ‘The other RCTs evaluated various interventions delivered by parents and found inconsistent or negative results. Studies were very small (most enrolled 20-40 children), and study quality was generally fair’ [8].

The review authors concluded that ‘among the behavioural interventions, those based on applied behaviour analysis have the highest-quality data supporting their effects on cognitive and language outcomes’ [7, 8].

The Cochrane review conducted by Reichow et al. assessed the effectiveness of EIBI in increasing functional behaviours and skills, decreasing autism severity, and improving intelligence and communication skills for young children with ASD [14]. EIBI is one of the most well-established interventions for children with ASD, and the most often studied one for this age group. The intervention is delivered 20 to 40 h weekly during several years. The review authors conducted the literature search up to August 2017 and included five studies (one RCT and four controlled clinical trials) that compared children under six years of age at the start of the treatment who received EIBI (*n* = 116) with children with no-treatment or treatment-as-usual control condition (*n* = 103). Three studies were conducted in the US, and the other two in the UK. The age of the children ranged between 30.2 and 40.5 months, and treatment duration was 24 to 36 months. They found at post-treatment that:

- EIBI may improve adaptive behaviour, assessed with the Vineland Adaptive Behaviour Scale (VABS) Composite, with a mean difference (MD) of 9.58 (95% CI: 5.57 to 13.60; 5 studies, 202 participants; low-quality evidence; lower values indicate positive effects).
- EIBI was not associated with any improvement in autism symptom severity (standardised MD − 0.34, 95% CI − 0.79 to 0.11; 2 studies, 81 participants; very low-quality evidence).
- EIBI may improve intelligence quotient (IQ), assessed by standardized IQ tests, with a MD of 15.44 (95% CI 9.29 to 21.59; 5 studies, 202 participants; low-quality evidence)
- EIBI may improve expressive language skills (SMD 0.51, 95% CI 0.12 to 0.90; 4 studies, 165 participants; low-quality evidence)
- EIBI may improve receptive language skills (SMD 0.55, 95% CI 0.23 to 0.87; 4 studies, 164 participants; low-quality evidence)
- EIBI was not associated with any improvement in problem behaviour (SMD − 0.58, 95% CI − 1.24 to 0.07; 2 studies, 67 participants; very low-quality evidence).

The Cochrane review authors concluded that ‘there is weak evidence that EIBI may be an effective behavioural treatment for some children with ASD’ and specified that the strength of the evidence was limited because the evidence ‘mostly comes from small studies that are not of the optimum design’ [14].

### Benefits of screening and early intervention

McPheeters et al. found no studies that directly compared ASD screening versus no screening in terms of short- and long-term health and social outcomes, including ‘improvements to core ASD symptoms, cognitive and intellectual functioning, language and communication skill development, challenging behavior, adaptive behavior, educational placement or achievement, or quality of life for the child and family’ [7, 8].

In addition, there are no studies that directly assess clinical outcomes of children identified with ASD through screening. Indeed, it is important to note that all the studies that assessed the effectiveness of interventions for ASD recruited children who were diagnosed with ASD based on developmental concerns raised by their family, teachers or clinicians. Therefore, most of the recruited children had significant impairments in cognition, language, and behaviour at the beginning of the interventions. However, the children detected by universal screening would be asymptomatic or present mild symptoms and are likely to be younger. ‘It is therefore not clear whether young children with ASD detected by screening and not because of parental or clinician concern will experience similar benefits from earlier intervention’ [7].

### Potential harms of ASD screening and interventions for children and their family

There are no studies assessing harms of ASD screening or harms of early interventions [3, 8].

Potential harms of universal screening for ASD include ‘misdiagnosis and the time, effort, and anxiety associated with further testing after a positive screening result’ [7]. ‘This is of particular concern when there is a delay in confirmatory testing because of resource limitations’, and ‘even good-quality studies of screening had a high dropout rate between screening steps and between screening and diagnosis, suggesting that the process may be difficult for some families’ [7, 8].

Potential harms of behavioural treatment were not considered to be significant but can be associated with important burden for the families in terms of time and resources [7]. The studies included in the Cochrane review reported no adverse effects associated with EIBI [14].

Overall, the USPSTF concluded that ‘the harms of screening for ASD and subsequent interventions are likely to be small based on evidence about the prevalence, accuracy of screening, and likelihood of minimal harms from behavioural interventions’ [7, 8].

## Summary of findings

There are discrepancies among the recommendations given by different institutions on universal screening for ASD in children.

- The Centers of Disease Control and Prevention and the American Academy of Pediatrics recommend that all children should be screened with an ASD-specific instrument during well-child visits at ages 18 and 24 months in conjunction with ongoing developmental surveillance and broadband developmental screening.
- The United States Preventive Services Task Force concludes that the current evidence is insufficient to assess the balance of benefits and harms of screening for ASD in young children for whom no concerns of ASD have been raised by their parents or a clinician.
- The PrevInfad workgroup (Spanish Association of Primary Care Pediatrics), the United Kingdom National Screening Committee and the Canadian Task Force on Preventive Healthcare recommend against universal screening, but for a screening among children with high risks.
- There is adequate evidence that ASD screening tools applied to children between 12 and 36 months accurately identify those with ASD.
- There is some evidence showing benefit of early interventions applied to children with ASD. However, this comes from small studies that recruited children who were clinically referred, those are children identified with developmental concern by their family, teacher or clinicians. We found no evidence on the effectiveness of interventions applied to children with ASD detected through screening.
- There is limited evidence assessing harms of screening or interventions for ASD. Potential harms derived from screening for ASD and subsequent interventions are likely to be small. However, interventions can be associated with important burden for the families in terms of time and resources.
- Overall, there is insufficient evidence to assess the balance of benefits and harms of universal ASD screening in young children.

## Data Availability

Not applicable.
